# Generative Artificial Intelligence in Health Research and Care: A Narrative Review of Applications and Implications for Alzheimer’s Disease and Related Dementia

**DOI:** 10.3390/bioengineering13101225

**Published:** 2026-10-20

**Authors:** Sam Omidi, Juanita-Dawne R. Bacsu, John Robert Absher, Faezeh Babaieasl, Hadi Kooshiar, S. Misagh Imani, Nafiseh Ebrahimi, Matthew Lee Smith, Zahra Rahemi

**Affiliations:** 1 School of Dentistry, Marquette University, Milwaukee, WI 53201, USA; sam.omidi@marquette.edu; 2 School of Nursing, Thompson Rivers University, Kamloops, BC V2C 0C8, Canada; jbacsu@tru.ca; 3 School of Medicine, University of South Carolina, Greenville, SC 29605, USA; absher@mailbox.sc.edu; 4 College of Nursing and Health Sciences, Texas A&M University, Corpus Christi, TX 78412, USA; faezeh.babaieasl@tamucc.edu (F.B.); hadi.kooshiar@tamucc.edu (H.K.); 5 Institute for Engineering in Medicine, Health, and Human Performance (EnMed), Batten College of Engineering and Technology, Old Dominion University, Norfolk, VA 23529, USA; simani@odu.edu; 6 School of Computing, College of Engineering, Computing and Applied Sciences, Clemson University, Clemson, SC 29634, USA; nebrahi@clemson.edu; 7 School of Public Health, Indiana University, Bloomington, IN 47405, USA; matlsmit@iu.edu; 8 School of Nursing, Clemson University, Clemson, SC 29634, USA

**Keywords:** generative artificial intelligence, large language models, Alzheimer’s disease, dementia, cognitive assessment, caregiver support

## Abstract

The advent of generative artificial intelligence (AI), particularly large language models (LLMs) and deep generative networks, is transforming the landscape of Alzheimer’s disease and related dementia (ADRD) research and care. This narrative review aims to synthesize current applications of generative AI in ADRD research and care, highlight ethical and implementation challenges, and propose strategies for responsible integration into clinical practice. We conducted a narrative review of literature, regulatory documents, program reports, and relevant sources published between 2016 and 2026, with a specific focus on cognitive screening, synthetic data generation, digital twin modeling, and caregiver support. Techniques discussed include generative adversarial networks (GANs), variational autoencoders (VAEs), diffusion models, multimodal transformers, and LLM-powered chatbots. Generative AI enables applications such as virtual patient companions, remote cognitive assessments via conversational agents, multimodal image-to-text tools, synthetic data augmentation, disease progression simulation, and improved caregiver communication. Challenges include model explainability, clinical validation, regulatory compliance, privacy, data bias, hallucination risk, patient autonomy, and global disparities. Responsible development requires transparent reporting, stakeholder co-design, rigorous clinical and economic validation, and continuous post-deployment monitoring to support fairness and effectiveness in real-world settings.

## 1. Introduction

Artificial intelligence (AI) and machine learning (ML) have significantly advanced Alzheimer’s disease and related dementia (ADRD) research and care over the past two decades. Initial applications focused on neuroimaging, where classical and deep learning (DL) models such as support vector machines (SVMs) and convolutional neural networks (CNNs) achieved strong diagnostic performance and helped identify imaging biomarkers associated with disease progression [1,2,3]. More recently, ML has enabled the integration of multiple data types, including genetic risk (e.g., APOE), laboratory values, cognitive assessments, and demographic information, supporting personalized risk prediction and modeling of disease trajectories, although ML does not always outperform conventional statistical models [4,5].

AI-based analysis of speech, language, and wearable sensor data has improved early detection of ADRD [6]. ML risk models can capture complex and nonlinear relationships among risk factors and, in some studies, have outperformed conventional approaches [2,7]. However, challenges such as limited model interpretability, data variability, and small training datasets, especially for underrepresented populations, continue to hinder clinical adoption [3,8].

The emergence of generative AI and large language models (LLMs), such as ChatGPT (OpenAI, San Francisco, CA, USA), marks a major shift in ADRD research and care. Unlike traditional models that classify or predict, LLMs generate original content, synthesize information, and support human-like dialogue, enabling applications in literature review, research summarization, and workflow optimization [9,10]. Deep generative models, including generative adversarial networks (GANs) and variational autoencoders (VAEs), are increasingly used to create synthetic datasets and advance drug development [11,12,13]. Moreover, conversational agents are gaining attention for their potential to engage older adults, patients, and caregivers through natural language interaction [14].

Most existing literature reviews focus on conventional ML and DL methods for ADRD diagnosis, often overlooking recent developments in generative AI, LLMs, and patient-centered technologies [1,7,8,15]. For instance, Zhao et al. [16] reviewed conventional ML and DL approaches for Alzheimer’s disease (AD) classification based on magnetic resonance imaging (MRI). Also, Alsubaie et al. [17] systematically reviewed DL models for AD detection from neuroimaging. While comprehensive within their scope, both concentrate on diagnostic classification from imaging data and do not address generative AI, LLMs, or patient- and caregiver-facing applications. The rapid rise of tools like ChatGPT, along with ongoing discussions around regulatory, ethical, and clinical implications, highlights the need for a timely and comprehensive review. This paper examines the role of generative AI and LLMs in ADRD research and care, emphasizing emerging applications, ethical and legal challenges, and their integration into clinical workflows and daily life. We aim to provide practical guidance for researchers, clinicians, and policymakers working to responsibly apply generative AI to improve outcomes in ADRD care.

Several reviews of AI in dementia have been published since 2023, but each covers only one specific part of the field [18,19,20]. To the best of our knowledge, no review has yet brought together the full spectrum of generative AI in ADRD, from LLM-based screening, patient companionship, and caregiver support to deep generative models for synthetic data, multimodal integration, and digital twins, while also examining the ethical, regulatory, and implementation challenges that determine whether these tools can be used safely in practice. This review was designed to fill that gap by offering an integrated, critical synthesis of the current evidence, highlighting both the opportunities and the limitations of generative AI, and identifying priorities for researchers, clinicians, and policymakers working toward its responsible use in ADRD research and care.

### Review Approach and Search Strategy

In this narrative review, we searched PubMed, Scopus, and Google Scholar for records published between 2016 and 2026, combining population terms (“Alzheimer*”, “dementia”, “mild cognitive impairment”, “caregiver*”) with method terms (“generative AI”, “large language model*”, “ChatGPT”, “chatbot”, “conversational agent”, “generative adversarial network*”, “variational autoencoder*”, “diffusion model*”, “synthetic data”, “digital twin”) and governance terms (“regulation”, “FDA”, “AI Act”, “MHRA”, “validation”, “bias”, “cost-effectiveness”). Reference lists of included articles and prior reviews were hand-searched, and regulatory documents, program reports, and relevant webpages were retrieved from the websites of the U.S. Food and Drug Administration (FDA), the European Commission, the UK Medicines and Healthcare products Regulatory Agency (MHRA), and the World Health Organization (WHO). Relevant company press releases were also consulted for product-specific updates. We included English-language empirical studies, reviews, conference papers, books, guidelines, regulatory documents, and preprints on generative AI in ADRD research or care, prioritizing sources from 2022 onward for LLM-specific evidence. These terms guided the main searches, while additional topic-specific searches were conducted to address methodological, ethical, regulatory, and implementation issues. Relevant foundational and cross-cutting sources were also included, even when they were not specific to ADRD.

## 2. Background: From Conventional AI to Generative Models in ADRD

Early applications of AI in ADRD focused on supervised learning and DL, particularly using neuroimaging data. Classical models such as SVMs and random forests were trained on handcrafted features derived from MRI and positron emission tomography (PET) scans, achieving strong performance in distinguishing ADRD from normal aging [16]. The introduction of CNNs marked a key shift. Deep learning models trained on large datasets, such as the Alzheimer’s Disease Neuroimaging Initiative (ADNI), have reported classification accuracies of around 85–90%, although concerns about limited generalizability persist [21,22]. Combining imaging modalities, such as MRI and PET, has further improved diagnostic performance and supports differentiation between AD and other dementias [22,23].

Beyond neuroimaging, AI has also been applied to cognitive assessments, electronic health records (EHRs), and multimodal datasets. ML enables automated scoring and prognosis, while natural language processing facilitates non-invasive screening for cognitive decline [24,25,26]. However, clinical integration remains limited due to issues with generalizability, model interpretability, and regulatory barriers [8].

A substantial shift is occurring with the emergence of generative AI and LLMs. Unlike discriminative models, generative models, including LLMs, diffusion models, VAEs, and GANs, produce synthetic data, support natural language interaction, and facilitate biomedical knowledge generation [27,28]. Table 1 outlines key distinctions between traditional and generative AI methods in ADRD research and care. Integrating generative AI into ADRD research may help close persistent gaps in generalizability, interpretability, and clinical implementation.

## 3. LLMs for Cognitive Assessment, Patient Interaction, and Caregiver Support

### 3.1. Conversational Agents for Screening

LLM-powered chatbots have shown potential as virtual examiners for cognitive screening in ADRD. These systems can administer verbal tasks (e.g., fluency and recall) and analyze spontaneous speech for early signs of impairment, but the most frequently cited evidence must be read with its methodological context. Balamurali B.T. and Chen [29] evaluated ChatGPT-3.5, ChatGPT-4, and Bard (Google LLC, Mountain View, CA, USA) using two independent zero-shot prompts (Q1 and Q2) on transcripts generated using Otter.ai (Otter.ai, Inc., Mountain View, CA, USA) from 71 picture-description recordings from the test set of the 2021 Alzheimer’s Dementia Recognition through Spontaneous Speech only (ADReSSo) challenge (36 cognitively normal [CN], 35 AD), comparing predictions with the dataset’s AD/CN labels and categorizing responses as AD, CN, or “Unsure.” Averaged across the two prompts, overall accuracy was 52%, 38%, and 54%, respectively. The reported figures of 56% for GPT-4 identifying CN cases and 89% for Bard identifying AD cases represent class-specific recall rates. However, GPT-4 returned “Unsure” for 57.1% and 51.4% of AD recordings under Q1 and Q2, respectively, while Bard misclassified 58.3% and 63.9% of CN recordings as AD and correctly identified only 27.8% and 11.1% as CN. Preregistration was not reported. The researchers acknowledged that ADReSSo samples might have been included in the chatbots’ training data and that variation across accounts, machines, and assessment times was not systematically evaluated. Prompt engineering improves on this baseline: Wang et al. [30] evaluated five iteratively developed prompt designs with GPT-3.5 and GPT-4 using text dialogues from 174 DementiaBank participants. For the final GPT-4 model, a single 52-participant held-out test set yielded a sensitivity of 77.3%, specificity of 83.3%, and an area under the curve (AUC) of 0.80 for mild cognitive impairment (MCI) versus normal cognition, with performance decreasing from a training AUC of 0.91 to a test AUC of 0.80; confidence intervals and external validation were not reported.

Comparison with conventional methods on speech benchmarks shows where LLMs stand. On the test set of the 2020 edition of this challenge (ADReSS), the linguistic-feature baseline achieved 75.0% accuracy [31]; on the ADReSSo 2021 test set, GPT-3 embeddings with a classical classifier reached 80.3% [32], while last-layer fine-tuned BERT achieved an F1 score of 82.8, and zero-shot LLaMA (Meta Platforms, Inc., Menlo Park, CA, USA) and GPT-4o (OpenAI, San Francisco, CA, USA) models achieved F1 scores of 68.5–73.1, increasing to as high as 81.1 after fine-tuning [33]. On a separate MCI corpus with independent training, validation, and test splits, fine-tuned LLMs achieved F1 scores of 64.5–68.2, while an augmented fusion model reached 72.8 [33]. These results suggest that zero-shot prompting does not consistently outperform conventional linguistic approaches, whereas LLMs may provide greater value as feature extractors, through task-specific fine-tuning, or for data augmentation. Automated systems such as the Mili platform (ki:elements GmbH, Saarbrücken, Germany) have also demonstrated the feasibility of remote follow-up cognitive testing, with 67 of 68 participants completing both visits in a study focused on usability and acceptability rather than diagnostic performance [34]. Representative tools are summarized in Table 2. No LLM-based screening method has yet been evaluated prospectively, with pre-registration, in a clinical screening population; existing tools should therefore be used only as adjuncts to clinical judgment, given their variability in outputs, sensitivity to prompting, and potential for misclassification.

### 3.2. Virtual Companions and Support Chatbots

LLMs are being developed as conversational companions to support daily interaction, cognitive stimulation, and emotional well-being among people living with ADRD. Tools such as MemoryCompanion illustrate how GPT-based dialogue can be integrated with personalized avatars and voice to create more familiar conversational agents for ADRD care, with intended applications in reminiscence therapy, daily communication, and emotional support [35]. Building on similar principles, a GPT-4-based conversational agent co-designed with clinical therapists, called the MindTalker system, was evaluated in eight people with early-stage dementia for about one month [36]. Participants valued the novelty of AI interaction but sought more consistent and meaningful engagement, stressing the irreplaceable value of human connection alongside AI companionship. These findings suggest that while LLM-powered companions can open new channels of social engagement for people with dementia, they must be carefully calibrated to complement rather than substitute human relationships [36].

**Table 2:** Representative examples of LLM applications in ADRD screening, patient support, and caregiver aid.

System/Study	AI Model	Use Case	Benefits	Limitations
Screening of MCI [30]	GPT-4 (prompt-engineered)	Classifies transcripts as MCI vs. normal	Sensitivity 77%, specificity 83%, AUC 0.80 (hold-out *n* = 52); scalable	Not a diagnostic substitute; risk of false results
ChatGPT/Bard ADRD Detect [29]	ChatGPT-3.5, GPT-4, Bard	Identifies ADRD via speech text	Zero-shot learning; flags language patterns	Overall accuracy 38–54% (*n* = 71); high “unsure” and false-positive rates; not replicated
MemoryCompanion [35]	Fine-tuned GPT-3.5 Turbo w/avatar/voice	Virtual daily companion, reminiscence	Continuous companionship; aims to reduce loneliness	Not trial-validated; privacy/safety concerns
ADQueryAid [37]	GPT-4 + RAG	Caregiver Q & A, advice, emotional support	Grounded in curated content, empathetic; usability 83.8 vs. 72.5 (*n* = 20)	Needs curation; risk of over-reliance
CareHeroes App [38]	Chatbot in mobile app	Tracks patient/ caregiver data, offers tips	High engagement; lower depression at 3 months (*n* = 12 analyzed of 21)	Pilot-only; basic chatbot features

RAG, retrieval-augmented generation.

Community-based programs such as Life Story Club use structured life-story conversations, delivered by phone or video aim to reduce loneliness and support psychosocial well-being among older adults [39]. AI-based systems can deliver empathetic dialogue and reminders, potentially enhancing quality of life and reducing caregiver burden [35,37]. More recently, scalable AI-driven approaches, such as the AI-CONECT chatbot, built on the I-CONECT clinical trial protocol, aim to extend the reach of cognitively stimulating conversational interventions to older adults with MCI, overcoming the human resource limitations inherent in traditional one-on-one programs [40]. Collectively, these systems point toward a future in which LLM companions serve as information resources and sustained social presences, capable of delivering reminiscence prompts, daily check-ins, and adaptive emotional support.

Despite their promise, these tools carry significant risks that are not yet adequately addressed in the literature. Conversational applications that offer companionship to combat loneliness risk exacerbating social isolation, as overreliance on AI may come at the expense of real-world social activities. Moreover, LLM hallucinations, which involve the generation of factually incorrect or fabricated content, present a particular hazard in ADRD care. People with dementia are especially vulnerable to such errors, given their impaired capacity to critically evaluate AI-generated content. In addition, their effectiveness remains unproven in large-scale randomized trials, while questions surrounding informed consent, data ownership, and emotional dependency are only beginning to receive scholarly attention [18]. User trust and cognitive safety must therefore serve as foundational design principles in the continued development of LLM-based companions for ADRD care [41].

Quantitative evidence on longer use comes from adults recruited online: in a four-week randomized study of 981 adults, longer daily ChatGPT use was associated with greater loneliness (β = 0.02, *p* = 0.027), lower socialization (β = −0.05, *p* = 0.0019), and greater emotional dependence (β = 0.06, *p* < 0.001); no significant overall effects of interaction mode were detected [42]. Overvalidation and sycophancy may reinforce delusional beliefs and distorted perceptions of reality, while cognitive impairment may affect how users interpret a system’s agency, reliability, and boundaries [43]. Non-deception disclosure, usage limits, escalation to humans, and monitoring for dependence should therefore be design requirements for LLM companions in ADRD.

### 3.3. LLMs for Caregiver Support

LLM-based tools are being developed to support caregivers of individuals with ADRD by offering real-time, personalized guidance and emotional assistance. ADQueryAid, for instance, combines GPT-4 with RAG over curated Alzheimer’s Association content; in a study with 20 caregivers, it scored 83.8 versus 72.5 for ChatGPT-3.5 on the Chatbot Usability Questionnaire, although outcomes were self-reported and the prototype was not HIPAA-compliant [37]. Apps like CareHeroes integrate chatbots to monitor well-being, provide advice, and offer positive reinforcement. In a usability pilot with 21 caregivers from two memory clinics, the chatbot was the most used feature, and depression scores were lower at three months (t11 = 2.03, *p* = 0.03)—a feasibility signal from roughly a dozen analyzable participants, not evidence of efficacy [38]. General-purpose models help but fall short in consistent ways: when ChatGPT-3.5 answered 60 real caregiver questions posted on Reddit, an interprofessional panel rated 78% of responses 4 or 5 out of 5, but comprehensiveness was lowest (63%), and the model sometimes suggested strategies the caregiver had already tried [44]; against Google Search (Google LLC, Mountain View, CA, USA) on 60 dementia queries, ChatGPT was more often highly relevant (97% of responses) but cited no sources and wrote at a mean Flesch–Kincaid grade level of 12.2, well above an eighth-grade reading level [45]. Custom systems grounded in curated content improve usability and perceived empathy, but none has yet shown better accuracy or safety than general-purpose models head-to-head.

### 3.4. Interface Modality, Research Thrusts, and Frontiers

How an LLM is delivered—as text on a screen, through a voice interface, or embodied in a social robot—shapes who can use it and how it fails. Screen-based chatbots dominate caregiver-facing research and are easiest to ground and audit, but they presuppose literacy and vision and produce text at about a 12th-grade reading level [45]; in testing of the culturally tailored Lola tool, caregivers with low vision or limited computer experience preferred voice, whereas hearing-impaired users were frustrated by audio-only output [46]. Voice offers an accessible mode of interaction but is vulnerable to speech-recognition errors: in a benchmark of voice-assistant commands modeled on Alexa (Amazon.com, Inc., Seattle, WA, USA) from 83 older adults, the word error rate of Whisper (OpenAI, San Francisco, CA, USA) averaged 0.21 for speakers with dementia versus approximately 0.08 for CN speakers, while no significant difference was observed between MCI and CN speakers [47], and commercial systems evaluated in a 2020 study had nearly twice the word error rate for Black speakers as for White speakers [48]; because recognition errors can affect downstream inference, voice agents may face greater usability barriers in dementia than in MCI. LLM-powered embodied agents may add social presence but have limited controlled evidence: a 2026 systematic review of 40 conversational-AI studies in dementia and MCI care (eight randomized trials) found promising evidence for low-cost text and telephone modalities, reported that a GPT-3.5 upgrade for long-term care residents increased mean session length approximately 18-fold with no adequately powered LLM trial against usual care identified in the review, and identified speech recognition as the most consistent technical barrier [20]; social and companion robots raise concerns about deception and attachment [49].

The LLM literature thus falls into five research thrusts with distinct frontiers: (1) speech- and language-based screening, where the frontier is prospective, pre-registered validation in screening populations and multilingual corpora; (2) patient-facing companionship, where feasibility has been shown in small pilots and the frontier is controlled trials with validated loneliness and cognitive outcomes; (3) caregiver information and coaching, where retrieval-grounded systems have shown promising usability and the frontier is demonstrated gains in accuracy, safety, readability, and caregiver outcomes; (4) modality and embodiment, where the frontier is dementia-robust speech recognition, redundant voice-plus-text interfaces, and LLM dialogue on robot platforms that already have trial evidence; and (5) safety and evaluation science, where longer daily use has been associated with adverse psychosocial outcomes in adults [42] and the frontier is dementia-specific exposure limits, non-deception standards, and blinded, longer designs reported using the TRIPOD-LLM guideline (Transparent Reporting of a multivariable model for Individual Prognosis Or Diagnosis—Large Language Models) [50]. Table 3 summarizes the screening studies; none is prospective, pre-registered, or externally validated in an independent clinical cohort.

## 4. Generative Models for Data Augmentation and Multimodal Integration

### 4.1. Synthetic Data Generation

Generative models, such as GANs, VAEs, and LLMs, are increasingly used in ADRD research to create synthetic data, addressing challenges such as data scarcity, imbalance, and privacy constraints. In neuroimaging, GANs generate realistic MRI and PET scans, supporting data augmentation and cross-modality translation (e.g., MRI-to-PET), thereby improving model performance and balancing datasets [51,52]. Reported gains vary with study design: the DeepCGAN study applied a convolutional GAN to sequences of ADNI clinical and cognitive features (not images) and reported 97.3% accuracy, but on a single 80/20 split without external validation or stated subject-level separation [53]. By contrast, a VAE pipeline with subject-level splits achieved 75% accuracy for AD versus CN on ADNI and 86% on an external Open Access Series of Imaging Studies (OASIS) cohort [54], and a systematic review of 14 GAN studies (11 meta-analyzed) found better pooled performance than non-GAN methods for AD versus CN (AUC 0.96 vs. 0.93) but not for progressive versus stable MCI (odds ratio 1.15, *p* = 0.31) [22]. VAEs, while less common, model latent patient features for classification and controlled sample generation [54,55].

LLMs can generate synthetic clinical text, such as patient notes, test transcripts, or symptom narratives. LLMs prompted with a few examples can extract ADRD phenotypes from clinical notes [56], and LLM-generated synthetic speech transcripts modestly improved transformer-based detection when added to real data (F1 83.3 to 85.7 at 2× augmentation, with no further gain at larger ratios) [33]. This technique may enable privacy-preserving data sharing, assuming proper safeguards against memorizing identifiable information [57,58].

Synthetic data in ADRD research carries several notable advantages. First, generative models help address class imbalance by producing samples for underrepresented groups, improving fairness and model robustness [59,60]. Second, synthetic data can reduce privacy risk and ease constraints on data sharing because records need not directly disclose protected health information, although re-identification risk and regulatory obligations remain [58,61]. Third, synthetic “what-if” cases support exploration of rare ADRD subtypes or counterfactual clinical paths, expanding research possibilities beyond what real-world datasets alone can offer [62,63].

Alongside these benefits, important challenges must be acknowledged. With respect to realism and fidelity, synthetic data often lacks the nuance of real data and may introduce artifacts or overlook subtle patterns [51,64]. Regarding bias amplification, models can replicate or worsen biases in source datasets, requiring careful oversight and bias mitigation [65,66]. Finally, evaluating data utility and ensuring alignment with real-world distributions is critical. At the same time, privacy safeguards must prevent re-identification and protect effective de-identification.

### 4.2. Research Thrusts, Case Studies, and the Frontier for Deep Generative Models

Beyond augmentation, GANs and their successors have been applied in ADRD along several distinct thrusts. Cross-modality synthesis has shown promise: a 3D reversible GAN developed using data from 1086 ADNI subjects produced synthetic PET that, when combined with real MRI, supported AD-versus-CN accuracy of 89.1% versus 89.3% with real MRI and PET, although synthetic PET reflects structure-correlated patterns rather than true metabolic change [67], and structural-to-functional translation in 986 subjects reproduced known AD connectivity differences (Pearson correlation 0.707 ± 0.004; five-fold cross-validation) [52]. Restoration of short-acquisition PET has shown clinical feasibility using a generator conditioned on real PET input: a GAN that generated 20 min-like florbetaben PET images from 2 min acquisitions, trained on 236 patients, achieved standardized uptake value ratio (SUVR) correlations of approximately 0.99 with full acquisitions in temporal validation (*n* = 28) and external ADNI validation (*n* = 58; 5 min inputs), while mean intra-reader agreement for brain amyloid plaque load (BAPL) scoring in the internal validation set was high (weighted κ = 0.88) [68]. Other thrusts—progression simulation, scanner harmonization, and counterfactual explanation—are discussed elsewhere [27,51,69].

The frontier is the evaluation of diffusion models as alternatives to GANs. On brain MRI, a least-squares GAN (LSGAN) produced near-identical samples (multi-scale structural similarity index measure [SSIM] 0.9997 versus 0.654 for real images)—mode collapse—whereas a latent diffusion model trained on 31,740 UK Biobank volumes closely matched real-image diversity [70]; on identical ADNI data, a pathology-aware diffusion model synthesizing [18F]fluorodeoxyglucose (FDG)-PET from MRI and clinical data achieved a peak signal-to-noise ratio of 24.6 dB versus 16.4 for CycleGAN and improved balanced accuracy for AD by four percentage points over MRI alone, without an external cohort and with smoother generated images and less pronounced pathology [71]. These gains come with sampler-dependent computational costs (142 s versus 7.6 s per 3D volume for 1000-step sampling versus 50-step denoising diffusion implicit model (DDIM) sampling, respectively [70]) and potentially higher privacy risk: unconditional latent diffusion models reproduced near-copies of training images in approximately 68.7% of samples on average across the evaluated medical imaging datasets, with larger training sets reducing memorization [72]. Memorization audits, external validation across scanners and populations, and clinician reads of synthesized PET should therefore be prerequisites for sharing synthetic ADRD data.

### 4.3. Multimodal Generative AI

ADRD involves diverse data types, neuroimaging, clinical text, and biomarkers, making multimodal integration essential. Multimodal generative AI, particularly vision-language transformers, enables unified processing and generation across modalities [73,74,75]. Recent models align MRI features with clinical language to support cross-modal tasks such as retrieval and captioning. For instance, dual-encoder systems can retrieve brain scans from text prompts (e.g., age, sex, and diagnosis) and support zero-shot ADRD classification [75,76]. In chest radiography, vision-language models have shown promising performance in report generation [77,78].

Medical image synthesis is also emerging, with diffusion models and GAN-based approaches generating synthetic brain MRIs from structured input [70], while recent text-guided diffusion models have been applied to ADRD-related tau PET synthesis [79]. These approaches may support data augmentation and the simulation of underrepresented disease patterns, although they remain experimental. Advanced multimodal transformers integrate imaging, clinical, and genetic data for diagnosis and prediction of MCI-to-AD conversion [80].

### 4.4. Digital Twins and Simulation

Digital twins, which are virtual and dynamic models of individual patients, offer transformative potential in ADRD research and care by integrating multimodal data to simulate disease progression, predict outcomes, and personalize treatment [4]. Unsupervised generative models, such as Conditional Restricted Boltzmann Machines (CRBMs), can simulate full patient trajectories, producing cognitive and biomarker data that are statistically consistent with real longitudinal records. The foundational model was trained on 1909 participants pooled from the placebo arms of 28 trials and reproduced the correlation structure of real data under five-fold cross-validation (R^2^ 0.82–0.91) [4], but individual-level prediction of 18-month change remains modest, and in trials the method is used not as a replacement control arm but as a prognostic covariate (PROCOVA), qualified by the European Medicines Agency (EMA) in 2022; sponsor re-analyses report sample-size savings of roughly 9–15%, whereas an independent, non-generative nearest-neighbor approach achieved comparable savings—suggesting that the gain derives from prognostic statistics rather than generative fidelity [81,82]. Additionally, digital twin and disease progression models have been used to simulate interventions, such as drug treatment at the patient or cellular level, and to characterize patient subgroups that may respond differently [83,84,85].

### 4.5. Comparative Assessment of Model Families and Balance of Evidence

Table 4 compares the main model families on strengths, weaknesses, computational and data requirements, and clinical readiness. Across the evidence, generative AI has shown benefits when the task is imputation or restoration conditioned on a real input [52,67,68], when limited training data are augmented with synthetic samples [22,33], when an LLM serves as a feature extractor or fine-tuned classifier rather than a zero-shot oracle [32,33], and in caregiver-facing usability when outputs are grounded in curated content [37]. The available evidence does not demonstrate consistent superiority over existing methods for discriminating progressive from stable MCI [22], for zero-shot screening in the cited evaluations [29,33], for generalization across populations or languages (Section 8), and for caregiver-chatbot accuracy and safety [37,44]. The cited studies do not demonstrate improved patient outcomes in randomized trials (Section 7).

## 5. Bias, Fairness, and Ethical Challenges in Generative AI for ADRD

Generative AI offers great promise for advancing ADRD research and care, but it also raises serious ethical issues. Key concerns include data and model bias, LLM hallucinations (i.e., the generation of inaccurate, misleading, or fabricated outputs that are not grounded in factual data), risks to patient autonomy and privacy, and impacts on trust and mental well-being.

### 5.1. Bias in Data and Models

Bias in generative AI arises from unrepresentative training data and flawed models. Most LLMs rely predominantly on data from Western, English-speaking, and developed nations, limiting their accuracy for other populations [86,87]. Bias in AI systems is also evident in speech technologies. For example, a study of five commercial automated speech recognition systems found substantially higher word error rates for black speakers than for white speakers, with average error rates of 0.35 versus 0.19, respectively [48]. Similar concerns apply to cognitive tests such as the Mini-Mental State Examination (MMSE) and Montreal Cognitive Assessment (MoCA), which were originally developed for Western populations. Studies have shown that performance on these tests can be influenced by age, education, ethnicity, and language. This may reduce test accuracy in non-Caucasian patients and further worsen health disparities [88]. When generative AI systems are trained on, aligned with, or deployed alongside data and workflows that incorporate these assessments, they may inherit or amplify the same underlying cultural and demographic biases. LLMs trained on such biased data may reinforce stereotypes, recommending more invasive or psychiatric care for marginalized groups.

Mitigation involves diversifying training data, applying fairness-aware methods (such as data reweighting and adversarial debiasing), and conducting systematic bias audits throughout the AI lifecycle [89,90]. Stakeholder input and ongoing human oversight are essential for identifying and addressing biases [91]. AI systems that underperform for already-disadvantaged populations risk deepening existing health inequities, and developers, clinicians, and policymakers share a collective responsibility to prioritize fairness from the earliest stages of design through deployment [92].

### 5.2. Hallucinations and Misinformation

LLMs often generate hallucinations due to their probabilistic design and lack of internal fact-checking [93]. In healthcare, such errors can mislead patients or caregivers about symptoms, medications, or treatments, risking harm [94,95]. For example, an LLM might incorrectly claim no drug interactions or invent clinical trial data, delaying proper care.

To mitigate this, LLMs can be grounded in verified medical databases via RAG and paired with uncertainty estimation so that low-confidence answers are flagged and users are directed to a clinician, although LLMs’ self-reported (verbalized) confidence tends to be overconfident and less reliable than consistency-based measures [96,97]. Human-in-the-loop models, real-time fact-checking, and clear documentation of system capabilities and responsibilities also enhance safety.

### 5.3. Patient Autonomy and Privacy

AI-enabled care systems, including emerging generative AI applications, often rely on sensitive personal and health-related data, raising privacy and autonomy concerns, especially for cognitively impaired individuals who may not fully understand or consent to data use. Continuous monitoring via audio, video, or wearables can enhance safety but may intrude on dignity and autonomy. Data collected for care might be reused or shared without explicit consent, increasing ethical risks [98,99,100,101].

Privacy-preserving methods like federated learning and edge computing keep data local, reducing exposure [102,103]. Anonymization techniques, such as pitch-shifting voice data, help protect identity while enabling analysis [104]. Maintaining human oversight in decisions is essential to protect patient rights [105,106,107].

### 5.4. Trust and Psychological Impact

AI in ADRD care changes patient-caregiver dynamics, posing psychological and social risks. Overreliance on AI and automation bias may lead users to blindly trust AI, neglect personal judgment, or miss subtle clinical signs [108]. Patients or caregivers might delay medical attention if AI reassurances mask concerning symptoms. Caregivers and clinicians risk skill loss and disengagement from excessive dependence on AI.

Additionally, users of AI chatbots and companions may react negatively if AI-generated responses lack emotional sensitivity, and emotional dependence on such companions can cause harm [109,110]. Overuse of chatbots or social robots can increase loneliness if they replace human contact [42,49]. Safeguards include ethical AI design, maintaining human oversight for critical decisions, promoting AI as an augmentative, not a replacement, using empathetic language, providing clear disclaimers, and educating users about AI’s supportive role [100,111].

## 6. Transparency, Interpretability, and Explainability of Generative AI

Generative AI models like GANs, VAEs, and transformer-based LLMs offer powerful tools for ADRD research and care but pose transparency challenges. Their opacity hinders researchers, clinicians, and patients from fully grasping or trusting the models’ decisions, despite their ability to analyze complex neuroimaging, clinical, and language data [112,113,114,115].

A key challenge is the “black box” nature of these models. In transformers, outputs result from complex attention across layers and tokens, creating interdependencies that resist simple interpretation. Although attention maps highlight influential words or image regions, they do not provide causal explanations and may mislead users about interpretability [116,117]. For VAEs and GANs, internal representations lie in high-dimensional latent spaces that rarely align with clear clinical features. For example, a VAE encoding of an MRI scan may mix various morphological and textural traits without isolating features such as hippocampal atrophy [55,118,119]. Thus, understanding why a model generates a specific synthetic brain image or identifies linguistic markers of cognitive impairment remains difficult.

Stochasticity in generative models hinders transparency. These models sample from probability distributions, producing variable outputs even with identical inputs. Furthermore, generative models often lack ground truth outputs. Unlike classifiers evaluated against known labels, generative outputs can be plausible but unverifiable, which complicates post hoc explanation and validation [120].

Despite challenges, interpretability methods for generative models are advancing. Attention visualization is widely used in transformer-based language and vision models to highlight input elements, such as words in clinical transcripts or MRI regions, that influence outputs. In ADRD, attention maps may reveal reliance on linguistic features like pronoun use or pauses, which correlate with early cognitive decline [121].

Saliency and feature attribution methods such as SHapley Additive exPlanations (SHAP), Local Interpretable Model-agnostic Explanations (LIME), and Integrated Gradients (IG) provide quantitative interpretability by estimating each input feature’s contribution to a model’s output. In language-based dementia screening, they can reveal the influence of markers such as pronoun use or interjections on ADRD classification [122,123,124].

Visual explanation methods, such as gradient-weighted class activation mapping (Grad-CAM) and occlusion analysis, help localize key regions in synthetic or real neuroimages. For instance, heatmaps may show a classifier focusing on the medial temporal lobe or hippocampus, consistent with attention to clinically relevant areas [125].

Latent space exploration in VAE-based models can link hidden codes to diagnostic groups and disease-related brain patterns [55,126]. However, clinical interpretation remains difficult due to entangled features within latent variables.

Counterfactual explanations enhance interpretability by generating “what-if” scenarios. For example, a model’s output might be tested by reducing hesitations in a transcript or reducing the degree of hippocampal atrophy represented in an MRI. These perturbations help clinicians understand decision boundaries and AI robustness [69,127].

An emerging approach with LLMs is generating natural-language rationales for decisions. For example, after analyzing a patient’s narrative, an LLM could hypothetically explain, “The patient used nonspecific terms and significant pauses, linked to early ADRD.” This plain-language explanation would, in principle, improve clinician trust and support AI integration into diagnostics [128]. However, such explanations may oversimplify complex model reasoning or be inaccurate, potentially reflecting plausible but unfounded justifications rather than true underlying decision processes. Table 5 summarizes these interpretability methods and their clinical relevance for ADRD.

In ADRD research and care, interpretability is essential. Interpretable models are often preferred, frequently perform comparably to black-box models, and transparent approaches may improve trust [8,114]. Explainability also aids bias detection by revealing which features influence predictions, helping identify and correct demographic biases such as those related to education or dialect; such checks matter because models built on non-diverse reference data can produce systematically different errors across groups [129,130].

## 7. Validation and Regulatory Considerations for AI in ADRD Care

### 7.1. Clinical Validation Strategies

Validating generative AI in ADRD care requires more than technical accuracy. Although prospective clinical trials are the gold standard, they are rare for cognitive disorders [131,132]. Fewer than 2% of healthcare AI models move from prototypes to clinical use, raising doubts about their real-world impact [133]. Most dementia AI research remains retrospective, showing high accuracy on curated data but limited evidence of patient benefit in routine care. Systematic appraisals support the need for caution: of 81 non-randomized deep-learning imaging studies comparing AI with expert clinicians, only nine were prospective, six were tested in real clinical settings, and 72% were at high risk of bias [131], and none of the LLM screening studies in Table 3 is prospective, pre-registered, or externally validated in an independent clinical cohort.

A key validation step is benchmarking AI tools against established standards of care. AI-driven cognitive assessments, such as language analysis via LLMs or automated neuroimaging, should be directly compared with expert diagnoses in prospective trials [3,134]. Although rare, randomized controlled trials (RCTs) remain the most rigorous method for assessing the impact on clinical decisions and patient outcomes [131]. Evidence from RCTs of AI decision support systems shows that even highly accurate models may not improve outcomes when deployed in practice, highlighting that accuracy alone is insufficient [132]. For ADRD care, success means demonstrating earlier diagnosis, enhanced care planning, better patient quality of life, or more efficient use of resources and healthcare, not just high retrospective accuracy [15,135,136].

Real-world validation complements controlled trials, as AI systems often show performance drift or reduced utility across diverse patient groups and clinical settings. For instance, a dementia risk predictor with strong retrospective results may be rejected if it disrupts workflow or lacks usability or transparency [132]. Therefore, validation must include clinical impact measures such as diagnostic timeliness, reduced unnecessary testing or hospitalizations, cost savings, workflow integration, and clinician and patient acceptance. However, many AI RCTs overlook these implementation outcomes, with reviews revealing a lack of reporting on adoption, implementation costs, and sustainability [133].

Due to the scarcity of rigorous validation in dementia AI, regulatory bodies and clinical guideline panels remain cautious. Experts therefore advocate real-world validation and prospective studies that compare AI-augmented care with standard practice, in line with broader calls for diverse and representative participation in dementia research [137,138]. Table 6 summarizes the key validation pathways discussed in this section and outlines the description and associated challenges of each step for AI deployment in ADRD care.

### 7.2. Regulatory Landscape

Regulatory frameworks for medical AI are rapidly evolving across the United States (U.S.), European Union (EU), and United Kingdom (UK), each with distinct approaches. In the U.S., the FDA regulates most AI tools intended for diagnosis or treatment as Software as a Medical Device (SaMD), requiring safety, effectiveness, risk-based pre-market review, risk analysis, and post-market surveillance [139,140]. Manufacturers are encouraged to submit a Predetermined Change Control Plan (PCCP) to allow controlled updates without full re-approval for each software change [139,141]. Additionally, bias mitigation is increasingly emphasized, expecting developers to train and validate models on diverse populations to prevent worsening health disparities [142].

As of the FDA’s July 2025 list update, about 1250 AI-enabled devices had been authorized [143]. The ADRD pathway is illustrated by BrainSee (Darmiyan, Inc., San Francisco, CA, USA), which received De Novo authorization in January 2024 to aid assessment of five-year progression from amnestic MCI to Alzheimer’s dementia from routine MRI, cognitive scores, and demographic information; in May 2025, Darmiyan announced FDA approval of a seven-year post-market study of the tool in ethnically and socioeconomically diverse community populations—subgroup evaluation and performance monitoring built into an authorization [144]. In November 2025 the FDA stated that it had authorized no generative-AI device for mental health conditions, and its Digital Health Advisory Committee emphasized assessment of hallucination and bias risks, clear intended uses and limitations, escalation to humans, and post-market monitoring for performance drift and uncontrolled expansion of use [145]. Regulatory pathways therefore remain uncertain, particularly for general-purpose LLMs adapted for clinical applications.

In the EU, the Medical Device Regulation (MDR) and the recently enacted AI Act jointly regulate medical software; under the AI Act, AI systems that are, or are safety components of, medical devices requiring third-party conformity assessment are classified as “high-risk.” This classification demands strict risk management, data governance, transparency, and bias auditing throughout the product lifecycle [146,147]. The AI Act also requires human oversight, accuracy, robustness, cybersecurity, and ongoing post-market monitoring with incident reporting [146]. By requiring conformity assessments that address algorithmic transparency, bias, and robustness, the EU framework seeks to ensure that AI systems deployed in sensitive clinical contexts, such as dementia diagnosis, are held to the highest standards of accountability and safety [146].

The UK, post-Brexit, regulates AI-driven medical software through the MHRA within its evolving medical device regulatory framework, with AI-enabled medical devices generally treated as part of SaMD. This approach is supplemented by the UK Government’s pro-innovation AI regulatory strategy, which sets out five cross-sectoral principles, including safety, transparency, fairness, accountability, and contestability, while emphasizing agile, guidance-based, and proportionate oversight with clearer responsibility and opportunities for redress [148]. The UK approach currently combines regulatory reform, guidance development, and sandboxes, with an emphasis on evidence, transparency, and ongoing surveillance. The MHRA’s AI Airlock sandbox, launched in 2024, provides a regulator-run environment in which LLM-based products (generative radiology reporting, retrieval-augmented guideline assistants, ambient documentation) have been evaluated with regulator participation; its lessons emphasize retrieval grounding against hallucination, validation of synthetic data, and federated performance monitoring, alongside PCCP-style change control, but neither of the first two cohorts included a dedicated dementia or neurology product [149,150,151]. Comparative analyses across major jurisdictions point to a convergence of regulatory requirements around transparency, explainability, bias mitigation, and continuous surveillance, reflecting a shared recognition that public trust in clinical AI cannot be assumed [147].

### 7.3. Post-Market Monitoring and Updates

Regulatory approval marks only the start for AI in ADRD care. Continuous post-market monitoring is vital to maintain safety and effectiveness as models encounter new data, clinical practices, and patient populations [140,141]. Performance drift, declining model accuracy over time, is common, necessitating active systems to regularly assess accuracy, bias, and security. In the U.S., manufacturers are required to report adverse events involving medical devices, including AI-enabled devices, so that they can be investigated [152]. Model updates, whether intended to improve performance or mitigate bias, must be documented and validated and, when substantial, reported or re-certified.

### 7.4. Accountability and Post-Market Responsibility

Accountability is a key post-market issue. When errors occur, such as an LLM providing harmful advice or a diagnostic tool misclassifying cases, responsibility ultimately rests with both the clinician and the manufacturer. Current regulations emphasize maintaining a human in the loop for critical decisions, ensuring AI assists rather than replaces professional judgment [153,154]. Equally important is thorough user training, so clinicians understand AI’s capabilities, limitations, and oversight protocols, including clear reporting and remediation pathways for adverse events. Patients and caregivers should be informed when AI tools are involved in decision-making, including the systems’ roles and limitations, enabling informed consent and fostering trust. Table 7 presents a comprehensive checklist of validation and post-market monitoring requirements. It is intended to guide developers, clinicians, healthcare institutions, and regulatory bodies in the safe and effective deployment of AI systems in ADRD care. The checklist was developed by the author team by synthesizing regulatory principles and reporting guidelines for medical AI [50,139,140,141,142,146,147,155], and adapting them to the ADRD context. It has not yet been validated through a formal consensus process, which is a priority for future work.

### 7.5. Cost-Effectiveness, Reimbursement, and Resource Requirements

Economic evidence is limited in the generative-AI literature in ADRD. In the U.S., care for people aged 65 and older with dementia is projected to cost USD 409 billion in 2026, 55% of primary care physicians surveyed in 2019 reported a local shortage of dementia specialists, and unpaid caregivers provided 19.6 billion hours of care in 2025 [156]; the value of a triage tool therefore depends on diagnostic performance and downstream costs as well as its price. Formal evaluations are few: a 2025 systematic review included 19 economic studies, none focused on dementia or LLMs, often underreporting implementation and infrastructure costs [157]. Two dementia-specific models reach opposite conclusions: a manufacturer-associated UK analysis of an AI-scored cognitive test (GBP 21–32 per test, including staff time; specificity 80%) found it dominant over paper-based screening, saving GBP 123–226 per patient over a lifetime horizon [158], whereas an EU-funded early health-technology assessment of an AI risk-prediction tool for MCI found it dominated by usual care in its five-year base case at assumed sensitivity and specificity of 95%, because the modeled disutility of a high-risk label outweighed the benefit of a low-risk label, although cost-effectiveness varied with available treatments and the comparator [159]. Both models concern non-generative AI.

Reimbursement is application-specific: U.S. Medicare has reimbursed selected narrow-AI services through dedicated codes or temporary add-on payments, within existing payment structures [160]. Resource needs differ sharply by deployment mode: cloud inference costs depend on model choice and token volume, whereas privacy-driven on-premise deployment requires local computing resources whose capacity depends on model size, precision, and workload. Integration, validation, governance, and training costs must also be included in economic evaluations [157,160]. Cost-effectiveness and budget-impact evaluation should therefore accompany clinical validation (Table 8).

## 8. Global Perspectives and Societal Implications

Generative AI holds the promise of reducing ADRD care disparities globally, especially in low- and middle-income countries (LMICs) facing shortages of specialists and limited infrastructure. ADRD cases are rising rapidly in these regions, where patients often endure long waits or travel far for care [161,162]. Generative models deployed on common smartphones can help close these gaps by enabling remote cognitive assessment, triage, and support.

Innovative, low-cost AI applications are emerging that could expand access to ADRD care, although it is important to be precise about where they have been developed and tested. TapTalk, a smartphone test of hand movements and speech, is being developed in Australia as a preclinical screening tool; its device validation involved 31 healthy volunteers, no diagnostic accuracy has yet been published, and its suitability for low-literacy populations is argued but not yet shown [163,164]. Other digital tools, such as tablet-based screening apps used by lay health workers in local languages and chatbot- or voice-based information tools, may widen reach to underserved populations [165,166].

In principle, such platforms could host AI agents that assist families with early cognitive screening, medication reminders, or referrals; where early suspicion matters more than diagnostic precision, this could identify at-risk individuals who would otherwise be missed. This remains a hypothesis: a 2026 scoping review of AI for dementia diagnosis included 12 studies from high-income and three from upper-middle-income countries and none from low- or lower-middle-income countries [167]. The empirical evidence on cross-population transfer is instructive. In the multilingual ADReSS-M challenge, systems trained on English picture descriptions and tested on Greek reached 73.9% (baseline) to 87.0% (best team) accuracy, with eight Greek samples available for development, and disfluency features generalized across languages while differing picture content complicated evaluation of linguistic features [168]; English-trained speech markers applied without retraining to a matched Chilean Spanish cohort preserved timing-based discrimination (AUC 0.75), with weaker lexico-semantic discrimination (AUC 0.64; within-language 0.80) [169]. MRI classifiers trained on ADNI (83% White participants) transferred to a Korean cohort with a zero-shot MCI-class F1 of only 0.34, rising to 0.44 after fine-tuning on 30% of local data [170], and GPT-3.5 gave on average 18% fewer answers rated as comprehensive and appropriate on 2070 health queries in Spanish, Chinese, and Hindi than in English, with surface-level consistency falling by up to 50.7% in Hindi [171]. Transfer thus appears more robust for acoustic–temporal features and can improve after local fine-tuning but is less reliable for language-specific lexical content and some zero-shot applications across populations—raising concerns about the generalizability of text-based generative approaches.

Despite these opportunities, societal risks remain significant. Most advanced AI models, including LLMs like GPT-4, are primarily trained on English and Western data, limiting their cultural and linguistic relevance elsewhere [172,173]. When AI chatbots offer advice based on treatments unavailable in Africa or Asia, or when they misunderstand local dialects, they risk eroding trust or causing harm. De Vere Hunt et al. [174] emphasize the urgent need to develop local-language models, noting that current LLMs mostly cover European languages with Western cultural contexts, and point to open Swahili-language health LLMs, such as UlizaLlama (Jacaranda Health, Nairobi, Kenya), as a template.

Biases in data and model design have real-world impacts. For example, the Lola AI assistant for Black American Alzheimer’s caregivers highlighted that acceptability may depend on culturally resonant features, including voice chat, relevant visuals, and concerns about internet access and trust in technology [46,166].

Cultural adaptation extends beyond language. Generative AI must be co-designed with communities, integrating local norms, health practices, and priorities [175,176]. Voice-driven interfaces with familiar accents may reduce literacy barriers and democratize information access [166,175]. In many regions, basic infrastructure such as reliable power and internet cannot be assumed. Offline-capable apps, local data centers, or hybrid models that combine mobile chat with specialist input may be necessary, alongside investment in data infrastructure for AI research in LMICs [177,178].

The global research imbalance is stark. Although about 60% of people with dementia live in LMICs, most dementia research, including AI research, is concentrated in high-income countries [161,179]. Models developed on North American or European datasets may fail in Africa, Latin America, or Asia due to biological, social, or disease presentation differences. Addressing this demands investment in local research capacity, cross-regional collaboration across LMICs, and representative data collection [180]. Without such efforts, AI risks reinforcing global health inequities.

Equitable deployment involves not only adapting technology to local culture and language but also engaging local users in design, deployment, and evaluation, with clear oversight and redress mechanisms [137,181].

## 9. Limitations of This Review

This review has several limitations. First, it is narrative rather than systematic: we did not screen records in duplicate against pre-specified criteria, register a protocol, or assign formal risk-of-bias scores, so selection and citation bias cannot be excluded. Second, the primary literature consists largely of small, retrospective, non-pre-registered studies that recycle the same public corpora (156 ADReSS and 237 ADReSSo speakers; ADNI), so results are not independent and contamination of LLM training data by public benchmarks cannot be ruled out; our performance statements are internal-validation findings. Third, several cited studies are preprints or conference papers, model versions change within months, and regulatory facts are stated as of mid-2026. Fourth, our searches were restricted to English, and validated generative-AI dementia tools from low- and lower-middle-income countries do not yet exist, so our statements on global applicability rest on transfer studies rather than direct evidence and are conditional. Finally, because the checklist (Table 7) has not undergone validation via Delphi consensus, it should be utilized as a structured starting point.

## 10. Conclusions and Future Outlook

Generative AI in ADRD research and care is rapidly evolving, offering substantial opportunities alongside critical challenges. This review highlighted how deep generative models are being applied to ADRD patient education, risk screening, cognitive assessment, synthetic data generation, and care navigation and showed that the evidence is dominated by small, retrospective, internally validated studies: generative AI has shown benefits over existing methods mainly for missing-modality imputation, low-data augmentation, LLM-derived features, and caregiver-facing usability, whereas zero-shot LLM screening still trails fine-tuned language models, and, apart from the EMA qualification of a generative prognostic-covariate method for trial analysis, no generative tool has been prospectively validated, authorized as a medical device, or shown to be cost-effective in ADRD. At the same time, emerging ethical, regulatory, and societal concerns demand attention as AI integrates further into clinical and community settings.

These advances also raise issues of transparency, bias, privacy, safety, and the risk of inequitable access. Addressing fairness and issues of equity in access requires addressing linguistic and cultural biases, accessibility, trust, and rigorous oversight [137,182].

Innovations such as caregiver chatbots (e.g., the Lola app) and virtual assistants must demonstrate usability, trustworthiness, and cultural relevance to be effective [46,183].

Looking ahead, key priorities for the field are clear. First, publicly available, multimodal benchmark datasets are essential for developing and fairly evaluating generative models in ADRD. These datasets must represent global patient diversity across languages and clinical contexts. Second, prospective clinical trials and pragmatic studies of LLM- and generative AI-based tools are needed to move beyond retrospective accuracy toward real-world effectiveness and user acceptance. Third, participatory co-design should be standard practice, with patients and caregivers engaged throughout development to mitigate risks, reveal blind spots, and build trust. Fourth, interdisciplinary teams spanning clinical neuroscience, computer science, ethics, social science, and patient advocacy must become the norm to address the multifaceted challenges of generative AI in health. Fifth, economic evaluation—cost-effectiveness, budget impact, and reimbursement pathways—must accompany clinical validation, because we identified no payer that reimburses an LLM-based service and no trial of generative AI in ADRD has reported costs. Table 8 consolidates these priorities into actionable recommendations across three domains, including research, clinical practice, and policy/ethics, intended to guide the responsible and equitable advancement of generative AI in ADRD care.

Models should be trained and validated on data reflecting real-world diversity, not only in ethnicity and language but also in healthcare settings, literacy, and cultural norms. Investment in collecting underrepresented data, evaluating equity outcomes, and strengthening local research capacity is vital, as emphasized in emerging policy and regulatory frameworks [92,137].

In conclusion, generative AI holds substantial, though as yet largely unrealized, potential for ADRD care worldwide by democratizing access to expertise, personalizing interventions, and supporting families and clinicians. Realizing this vision demands vigilant ethical oversight, rigorous evidence, and sustained global collaboration.

## Figures and Tables

**Table 1:** Conventional versus generative AI approaches in ADRD.

Aspect	Conventional AI (ML/DL)	Generative AI (LLMs and Others)
Data Types Used	Structured data (e.g., MRI, PET, tabular EHR), often single modality, labeled datasets	Vast unstructured data (e.g., text, images, and audio), integrates multiple modalities, learns from raw data
Outputs	Predicted labels (e.g., diagnosis), risk scores, cluster assignments	Free-form text, synthetic data (e.g., MRIs and clinical notes), dialogue, reports, recommendations
Interpretability	Often opaque, needs post hoc explanation tools, rarely justifies predictions in natural language	More interpretable at the output level (e.g., natural-language explanations), though this does not equate to model-level transparency and remains subject to output accuracy limits
Validation	Well-studied, many retrospective studies, limited prospective trials or regulatory approvals	Emerging, mostly prototypes or proof-of-concept, real-world validation is ongoing
Example Use Cases	Classifying ADRD from controls using CNN on MRI, predicting conversion with cognitive test scores, EHR-based risk stratification	Generating synthetic MRIs, LLM chatbots for caregiver support, automatic literature reviews, AI-driven patient dialogue and note writing

**Table 3:** Design, validation, and reported performance of LLM-based cognitive-screening studies cited in Section 3 (companion and caregiver studies: see Table 2 and text).

Study [Ref.]	Task; Model; Dataset (*n*)	Reference Standard	Validation; Pre-Registration	Key Metrics; Independent Replication
Balamurali B.T. and Chen [29]	AD vs. CN; ChatGPT-3.5/GPT-4/Bard zero-shot; ADReSSo test set (*n* = 71)	Clinical diagnosis (Pitt corpus)	Single pass on test set; not pre-registered	Accuracy 52/38/54%; GPT-4 “unsure” 51–57%; Bard correctly classified only 11–28% of CN recordings; protocol not replicated
Wang et al. [30]	MCI vs. CN; GPT-4 prompt-engineered; DementiaBank (*n* = 174; test 52)	Database diagnosis (ECog, CDR, MMSE)	Single 70/30 split; no external set; not pre-registered	Sensitivity 77.3%, specificity 83.3%, AUC 0.80 (training 0.91); no CIs; not replicated
Agbavor and Liang [32]	AD vs. non-AD; GPT-3 embeddings + classifier; ADReSSo (train 166, test 71)	Clinical diagnosis	10-fold CV + hold-out; not pre-registered	Accuracy 80.3%; no external cohort
Zolnour et al. [33]	AD/MCI vs. CN; BERT ± LLM synthetic transcripts; zero-shot vs. fine-tuned LLMs; ADReSSo (*n* = 237) + separate MCI corpus (*n* = 205)	Clinical diagnosis	Train/validation/test + separate MCI corpus; not pre-registered	F1 83.3 → 85.7 (fusion model) with synthetic data; zero-shot F1 68–73 (LLaMA, GPT-4o); separate MCI corpus F1 65–73

CV, cross-validation; CI, confidence interval; CDR, Clinical Dementia Rating; ECog, Everyday Cognition; MMSE, Mini-Mental State Examination.

**Table 4:** Comparative assessment of generative model families used in ADRD research and care.

Model Family	Strengths	Weaknesses	Computational and Data Needs	Clinical Readiness in ADRD
LLMs (prompted, retrieval-augmented, or fine-tuned)	Few-shot text understanding; natural-language explanations; useful feature extractors	Hallucination; prompt sensitivity; benchmark contamination; grade ~12 readability; weaker in low-resource languages	Low per-query cloud cost; substantial GPU memory for on-premise large models	Pilots (*n* = 8–21); no prospective validation or authorization
GANs	Fast sampling; paired/unpaired translation; proven on 300–1000-subject cohorts	Mode collapse; instability; hallucinated texture	Single workstation GPU; hundreds of subjects	Research; closest to clinic for PET restoration and missing-modality imputation
VAEs	Stable training; structured latent space; good encoders	Blurry samples; entangled latents	Low; hundreds to low thousands of scans	Research; 75–86% accuracy with external validation
Diffusion/latent diffusion	Best fidelity and diversity; flexible conditioning; state of the art for MRI → PET	Slow iterative sampling; 3D memory pressure; highest memorization risk	Multi-GPU for 3D; ≈1000 paired subjects for translation, >10,000 volumes for unconditional 3D	Research, rapidly maturing; no external or clinician-read validation
CRBM/digital twins	Small tabular models; rigorous CV; regulatory engagement	Modest individual-level accuracy; sponsor-generated evidence	Low for tabular CRBM models; higher for imaging-based or multimodal twins; thousands of historical trial participants	Highest: EMA-qualified prognostic covariates in phase 2/3 analyses

CV, cross-validation; GPU, graphics processing unit.

**Table 5:** Interpretability methods, model scope, and clinical utility in ADRD.

Interpretability Method	Applicable Models	Clinical Relevance in ADRD
Attention maps (visualizing trans- former weights)	LLMs, vision transformers	Reveals important words, phrases, or image regions; supports trust in language/image analysis
Saliency and feature attribution (SHAP, IG, LIME)	Predictive models and the scoring components of generative pipelines (IG requires gradient access)	Quantifies each input feature’s impact; clarifies why a prediction or synthetic output was made
Grad-CAM, heatmaps for images	CNNs, GANs, VAEs	Localizes imaging regions most influential for a CNN classifier’s decision; validates focus on biomarkers
Latent space exploration/ disentanglement	VAEs, GANs, diffusion models	Links latent variables to clinical or biomarker features; helps simulate or interpret disease states
Counterfactual explanations	Any generative or perturbation-capable model	Shows what changes would alter the output; clarifies decision boundaries in language or imaging
Natural-language rationale generation	LLMs, hybrid systems	Provides human-interpretable explanations for outputs; supports clinical adoption and validation

**Table 6:** Clinical validation pathways and considerations for AI in ADRD care.

Validation Step	Description and Challenges
Prospective Clinical Trials	Rare but essential for demonstrating patient benefit. Should compare AI tools to standard-of-care and expert judgment.
Head-to-Head Benchmarking	Direct comparison with gold-standard cognitive tests or imaging protocols.
Real-World Implementation Studies	Test performance in diverse settings (clinics, nursing homes), capture usability and integration.
Multi-Dimensional Outcomes	Include measures such as timeliness, cost, patient satisfaction, and clinician acceptance, not just accuracy.
Continuous Feedback and Adaptation	Ongoing input from end-users to refine and adapt the AI tool in practice.

**Table 7:** Checklist for validation and post-market monitoring of ADRD AI.

Requirement	Description
Robust Clinical Validation	Prospective trial data, benchmarking against standard-of-care, and validation on diverse and multi-site populations.
Regulatory Compliance	Device classification, safety and effectiveness documentation, engagement with regulators, bias and cybersecurity safeguards.
Bias and Fairness Auditing	Evaluation across subgroups (age, sex, ethnicity, education), retraining as needed, transparent documentation.
Human Oversight and Training	Clear definition of the human clinician’s role, user training on tool limitations and appropriate use.
Auditability and Logging	Automatic, secure logs of AI operations and decisions; access for investigation and regulatory review.
Cybersecurity and Privacy	Regular security assessments, encryption, and compliance with health data laws (e.g., Health Insurance Portability and Accountability Act [HIPAA], General Data Protection Regulation [GDPR]).
Performance Monitoring	Ongoing measurement of accuracy, user feedback, drift detection, periodic re-validation, and version control.
Incident Reporting	Established process for reporting, investigating, and learning from errors or adverse events.

**Table 8:** Key priorities and recommendations for the future of generative AI in ADRD.

Theme	Recommendations
Research	Develop and share benchmark ADRD datasets (imaging, speech, text); evaluate generative models in real-world trials; involve patients and caregivers in co-design; foster interdisciplinary collaborations. Report sample size, reference standard, validation design, and pre-registration for every evaluation; audit synthetic data for memorization.
Clinical Practice	Pilot and validate AI tools in routine care; train end-users in AI literacy; incorporate transparency and explainability into clinical workflows; adapt tools to diverse languages and cultures.
Policy/Ethics	Mandate guidelines for data diversity and privacy; establish frameworks for AI transparency and accountability; invest in equitable deployment and local capacity; promote open sharing of models and data. Develop reimbursement pathways and require cost-effectiveness evidence; Delphi-validate ADRD-specific AI checklists.

## Data Availability

No new data were created or analyzed in this study. Data sharing is not applicable to this article.
